# A Classification-Based Blood–Brain Barrier Model: A Comparative Approach

**DOI:** 10.3390/ph18060773

**Published:** 2025-05-22

**Authors:** Ralph Saber, Sandy Rihana

**Affiliations:** 1 Department of Biomedical Engineering, School of Engineering, Holy Spirit University of Kaslik (USEK), Jounieh P.O. Box 446, Lebanon; 2 Centre de Recherche CHUM, Ecole PolyTechnique Montreal, Montreal, QC H3T 0A3, Canada

**Keywords:** blood–brain barrier, classification, machine learning, genetic algorithm, sequential feature selection, artificial intelligence, in silico modeling, drug discovery

## Abstract

**Background and Objectives**: Drug permeability across the blood–brain barrier (BBB) remains a significant challenge in drug discovery, prompting extensive efforts to develop in silico predictive models. Most existing models rely on molecular descriptors to characterize drug properties. Feature selection algorithms play a crucial role in identifying the most relevant descriptors, thereby enhancing prediction accuracy. **Methods**: In this study, we compare the effectiveness of sequential feature selection (SFS) and genetic algorithms (GAs) in optimizing descriptor selection for BBB permeability prediction. Five different classifiers were initially trained on a dataset using eight molecular descriptors. Each classifier was then retrained using the descriptors selected by SFS and GA separately. **Results**: The results indicate that the GA method outperformed SFS, leading to a higher prediction accuracy (96.23%) when combined with a support vector machine (SVM) classifier. Furthermore, the GA approach, utilizing a fitness function based on classifier performance, consistently improved prediction accuracy across all tested models, whereas SFS showed lower effectiveness. Additionally, this study highlights the critical role of polar surface area in determining drug permeability across the BBB. **Conclusions**: These findings suggest that genetic algorithms provide a more robust approach than sequential feature selection for identifying key molecular descriptors in BBB permeability prediction.

## 1. Introduction

The blood–brain barrier (BBB) is a physiological barrier that maintains brain homeostasis by controlling the exchange of molecules between the blood and the brain [1]. Consequently, the BBB blocks the passage of multiple molecules towards the brain, including administered drugs. This is beneficial when the target of the drug resides outside the brain since it prevents undesirable drug interactions and the ensuing phenotypic side effects. However, in the case of drugs targeting central nervous system (CNS) diseases, transport across the BBB is mandatory [2]. Therefore, the ability of drug candidates to cross the BBB has to be studied by all pharmaceutical companies during drug discovery. In this context, numerous in silico BBB models have been implemented by researchers in order to predict the behavior of drugs across the barrier [3]. These predictive models can be used during the early phases of drug discovery and hence allow companies to save time and money resulting from failures. Two different types of in silico BBB models exist in the literature: binary models, which aim at qualitatively predicting whether drugs cross the BBB (BBB+) or not (BBB-), and quantitative models, which attempt to qualify the permeability of the barrier to a given drug by computing the logarithm of the ratio of the concentration of the drug in the brain to that in blood (logBB) or its penetration rate (PR) [3]. In this context, K. Raja et al. [4] proposed two different stepwise regression models, one for the prediction of logBB values and the other for PR values. Other quantitative models are reviewed in [3]. Notably, paclitaxel, due to its high hydrophobicity, large molecular weight, and high number of hydrogen bond donors and acceptors, has been widely studied as a model compound in BBB permeability research. Its delivery via nanoparticle systems has also been extensively investigated to improve CNS accessibility [4]. While such models assign specific logBB/PR values for each drug, binary models have so far achieved a higher prediction accuracy and provide a preliminary insight regarding the behavior of candidate drugs, which is sufficient in the early drug discovery stages. Predominantly, the binarization of drug permeability across the BBB is performed by setting empirical thresholds to logBB values [5,6,7,8,9]. However, S. Kunwittaya et al. [6] have shown that varying logBB thresholds lead to a difference in the prediction accuracy. Therefore, binary BBB models based on logBB values are prone to biases introduced by threshold setting. On the other hand, Adenot and Lahana [10] introduced a dataset based on the activity of the drug in the CNS: if a drug is CNS-active, then it is necessarily BBB+. However, some drugs can cross the BBB but still show no activity in the CNS. Even though finding BBB- drugs based on CNS activity is consequently a challenging task, CNS activity-based datasets require no threshold setting and hence do not introduce the previously mentioned biases [10].

Machine learning is ubiquitously applied in the case of binary BBB models. In this context, different types of classifiers were trained in the literature including support vector machines (SVM) [6,8,11,12], linear discriminant analysis (LDA) [13], artificial neural networks (ANNs) [6] and multilayer perceptron (MLP) [8,9], k-nearest neighbor (kNN) [8], decision trees (DTs) [6,7] and random forests (RFs) [5,8,9]. Other studies have used consensus models, by training and combining multiple classifiers [8,9]. While consensus models mitigate the overfitting problem of single classifiers, they naturally require high computational power, especially when dealing with high-dimensional data.

The features used to train these classifiers are often molecular descriptors, which are chemical properties describing the drugs [3]. In some studies, the fingerprints of the molecules have also been added in order to reach better predictions [8,9,12]. On the other hand, novel approaches have used the drug side effects and indications for BBB penetration prediction [14]. These models achieved excellent prediction performance but relied on high-level phenotypes, which makes it difficult to extract significant biological explanations concerning drug interaction with the BBB.

Therefore, molecular descriptors remain the staple of classification-based BBB models. However, until today, the high dimensionality of the data based on molecular descriptors is still challenging. The selection of the most relevant features is crucial since it guarantees an improved prediction performance on the one hand and faster computation on the other by reducing the size of feature vectors. In order to study the effect of the chosen features on the classification performance, Y. Yuan et al. [12] compared the performance of SVM models trained by feature vectors containing different molecular descriptors, fingerprints, or a combination of both. Since trying all possible combinations of feature vectors dramatically increases the required computational time and power, an effective feature selection algorithm is needed. In this context, D. Zhang et al. [9] applied a genetic algorithm for the selection of the appropriate features and the optimization of SVM parameters. Nevertheless, choosing the most suitable algorithm for a given application is an important step since different algorithms may lead to convergence to different feature subsets and consequently affect the prediction results. Hence, this study compares the effect of the genetic algorithm (GA) method to that of sequential feature selection (SFS) on the different classifiers applied in the reported in silico BBB models.

## 2. Results

In this study, the first step was to train the classifiers without applying any type of feature selection algorithm. The results are tabulated in Table 1.

The highest accuracy value obtained with the test set when training the classifiers with all the initial features was 94.6%, achieved using the Artificial Neural Network (ANN) model, surpassing the 93.35% accuracy of the SVM with RBF kernel.

### 2.1. Feature Selection

**Figure 1:**
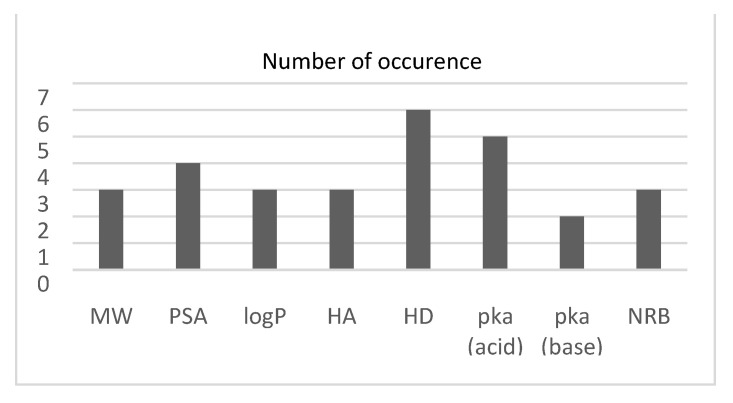
Histogram showing the number of times each descriptor was chosen using the SFS algorithm.

After obtaining the initial results, feature selection algorithms were performed, and the results are presented in Figure 1.

### 2.2. Part 1 Sequential Feature Selection

As described previously the convergence of the SFS towards the final feature subset is based on a criterion value extracted from the classifier itself: the number of misclassified observations in our study. Therefore, a specific feature subset was obtained with each classifier, as reported in Table 2.

As described previously, the convergence of the Sequential Feature Selection (SFS) algorithm toward the optimal feature subset is based on a criterion value derived from the classifier itself—in this case, the number of misclassified observations. Accordingly, a specific feature subset was selected for each classifier, depending on its sensitivity to different input combinations. The selected features for each model are summarized in Table 2. The feature subset selected for the ANN classifier emphasizes molecular descriptors related to size (MW), polarity (PSA), hydrogen bonding (HD), and ionization (pKa), reflecting the network′s sensitivity to both physicochemical and structural attributes. This tailored input configuration contributed to further improvements in classification accuracy when feature selection was applied.

### 2.3. Part 2 Genetic Algorithm

Two different genetic algorithms were used in this study, differing by the type of fitness value. In the first case, the fitness function returned the classification loss of an SVM, while in the next case, it returned that of a kNN. In the former case, the selected features were PSA and HD, whereas in the latter case, the selected features were PSA and pKa (strongest acidic).

#### Prediction Performance Evaluation

After running the feature selection algorithms, the same classifiers reported in Table 1 were trained separately using the selected features. The overall accuracy obtained on the test set is reported in Table 3.

In the case of SFS, the overall accuracy increased with the linear SVM as well as LDA, QDA, and kNN. The highest accuracy value reached was 94.98%, obtained with the QDA. On the other hand, the GA method resulted in an increase in the overall accuracy of all the classifiers in the case of a fitness function based on the classification loss of the SVM. The highest accuracy value reached was 96.23% with the SVM (polynomial kernel function). Nevertheless, with the kNN-based fitness function, the SVM (polynomial kernel) and the kNN witnessed a decrease in overall accuracy. The highest accuracy value was also 96.23%, obtained with the QDA, which was higher than that obtained with the SFS. Table 4 compares the performance of the two classifiers leading to the 96.23% overall accuracy in detail.

The integration of Artificial Neural Networks (ANNs) into the classifier comparison framework demonstrated consistently high accuracy across all configurations, both with and without feature selection. The ANN achieved 94.6% accuracy with all initial features, 95.51% after Sequential Feature Selection, Up to 96.04% with Genetic Algorithms (SVM-based fitness), nearly matching or exceeding the performance of classical models such as QDA and SVM.

These results reinforce the ANN′s ability to model complex, non-linear patterns in molecular descriptor space, positioning it as a robust tool for BBB permeability prediction.

**Figure 2:**
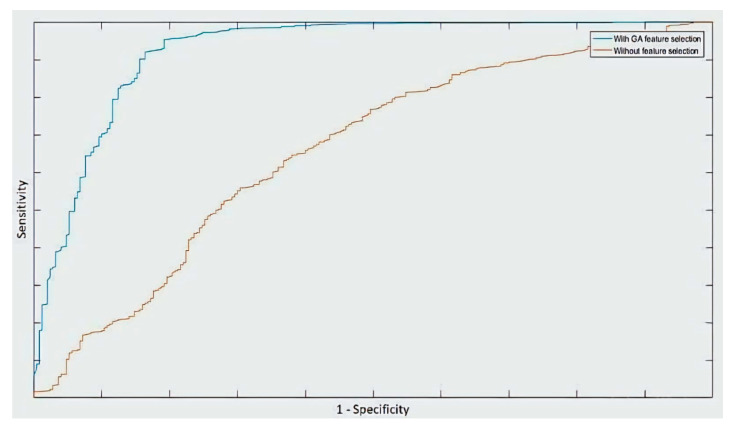
ROC curves of the SVM classifier (polynomial kernel) before (red) and after (blue) feature selection using the GA method.

Figure 2 illustrates the ROC curves of the SVM classifier trained with the entire feature set and after applying the GA method for feature selection. It is clear that the area under the curve is much higher after applying the GA method.

## 3. Discussion

The dataset used in this study was chosen since it is CNS-based [10], hence independent from logBB thresholds. The dataset was chosen to compare two types of feature selection algorithms, the backward sequential feature selection (SFS) and genetic algorithm (GA). In this study, the SFS resulted in larger feature subsets. At each iteration, SFS was used to perform 10-fold cross-validation, returning a criterion value while including other features. This is reflected by the given classifier. It should be noted that the number of hydrogen bond donors was selected with all the classifiers. This result is in line with the one reported in [15], which revealed that the hydrogen bonding characteristics are largely involved with the penetration across the BBB. However, the GA analysis showed that relying exclusively on the PSA and the number of hydrogen bond donors would lead to better results compared to the overall accuracy obtained (Table 3) as well as the ROC curve (Figure 2).

Moreover, it should be noted that the GA method (with SVM-based fitness function) led to an improvement in the results with all the reported classifiers, contrary to the SFS. The highest overall accuracy was also found with GA, and it had higher specificity than the SVM trained with the PSA and the HD. Nevertheless, both classifiers have a better balancing between predicting BBB+ and BBB- drugs than the binomial partial least squares implemented in [15] on the same data with selected molecular descriptors. Although the dataset used in this study does not include identifiable drug names for public disclosure, illustrative examples can highlight the implications of classification errors. A false positive (predicting BBB+ for a BBB− drug) may lead to unnecessary experimental validation and resource use. A false negative (predicting BBB− for a true BBB+ drug) could result in discarding a viable CNS-targeted candidate, potentially delaying innovative treatments. These misclassifications underline the importance of high sensitivity and specificity in in silico predictions.

## 4. Materials and Methods

**Figure 3:**
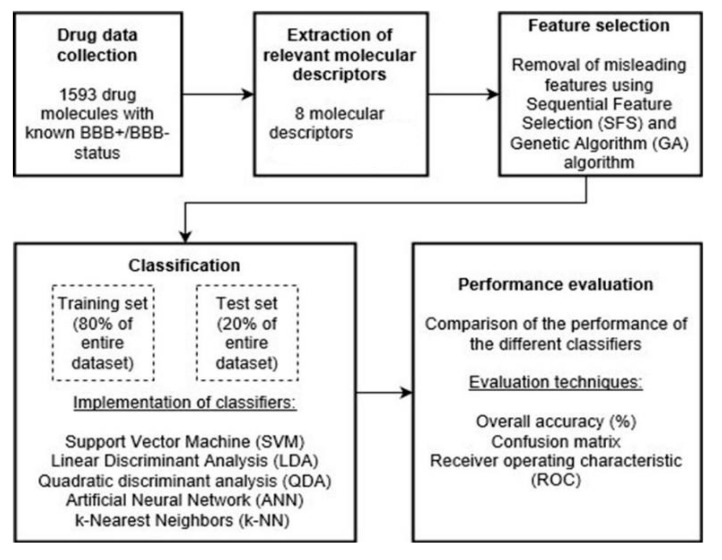
General workflow.

In order to compare the performance of GA to that of SFS, we first started by training and evaluating multiple classifiers without the application of any feature selection algorithm. Then, the same classifiers were implemented while applying each algorithm separately. The workflow including the use of feature selection is summarized in Figure 3.

### 4.1. Dataset Preparation

In this study, we chose to build and compare the models using the data made publicly available by [15]. The dataset is composed of 1593 drugs: 1283 that cross the BBB (BBB+) and 310 that do not (BBB). Since the authors used the dataset of Adenot and Lahana [10], the data were based on CNS activity.

### 4.2. Molecular Descriptors

Based on the correlation study performed by [15], 8 molecular descriptors were chosen in our work among the 19 descriptors reported in the dataset:

- The molecular weight (MW);
- The polar surface area (PSA);
- The octanol/water partition (logP);
- The number of hydrogen bond acceptors (HAs);
- The number of hydrogen bond donors (HDs);
- pka (strongest acid);
- pka (strongest base);
- The number of rotatable bonds (NRB).

### 4.3. Feature Selection

In order to evaluate and compare the performance of each algorithm, we separately applied each algorithm to the same dataset.

#### 4.3.1. Part 1 Sequential Feature Selection

This is an iterative algorithm that aims at finding the optimal combination of predictors that lead to the best prediction capacity of a specific classifier.

The algorithm may run in two opposite directions. On the one hand, it can start with the entire input features set and iteratively remove features that mislead a predefined classifier, until reaching the predictors’ subset, leading to the best classification performance; in this case, the algorithm is running in the backward direction. On the other hand, it may run in the forward direction by starting with an empty predictors’ subset and successively adding features that would improve the classifier’s predictive performance until reaching the optimal predictors’ subset.

**Figure 4:**
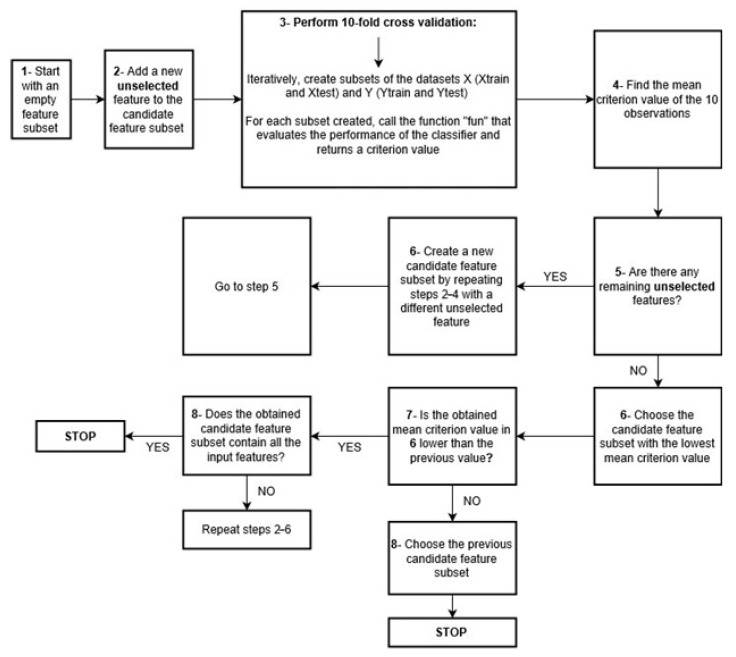
Flowchart of the forward sequential feature selection algorithm.

The steps of the forward algorithm are summarized in the flowchart presented in Figure 4. The algorithm starts by creating an empty feature subset. Then, it randomly adds one feature to the subset and performs 10-fold cross-validation, which returns a criterion value expressing the loss of the classifier. In this work, the criterion used by the algorithm for each combination of features was the number of misclassified observations in the test set. Then, the previously selected feature is removed, and a new feature is randomly added to the subset to find a new criterion value. Once all the features have been tried, the algorithm chooses the feature with the least criterion value as a permanent feature in the subset. Then, having this feature permanently present, the algorithm randomly adds a second feature to the subset, and a new criterion value is found. This is repeated until all the features have been tried. If the lowest criterion value of the feature subset (with two features) is smaller than the originally chosen subset (with one feature), the algorithm repeats the same steps by testing the addition of a third feature to the subset. Otherwise, the algorithm stops, and the previous feature subset is deemed the optimal one.

For example, if one needs to choose the optimal features from three initial ones, the algorithm successively calculates the criterion value obtained with each. If feature 1 leads to 0.056 as the criterion value, feature 2 yields 0.065, and feature 3 yields 0.078, the algorithm permanently chooses feature 1. Then, it tests the addition of feature 2 (classifier built with features 1 and 2) and feature 3 (classifier built with 1 and 3). If at least one of the two criterion values is less than 0.056 (initially obtained with feature 1), it tests the addition of the third feature to the feature subset. Otherwise, feature 1 is selected as the optimal feature.

#### 4.3.2. Part 2 Genetic Algorithm

Under the umbrella of feature selection, the genetic algorithm (GA) method was also applied with its label “the fittest survives” [16]. In fact, the GA method mimics genetic evolution by setting an initial population of binary chromosomes. Each gene is hence a binary digit in the chromosome. Afterward, at each new generation, the chromosomes undergo three different phenomena:

- Selection: the fittest chromosomes of the initial population are preserved for the next generation;
- Cross-over: new chromosomes are created in the new generation by mixing gene subsets of one chromosome with those of another;
- Mutation: A certain gene from a given chromosome is randomly inverted (0 to 1 or vice versa). This allows the algorithm to evaluate new options instead of getting stuck on local minima.

These phenomena are repeated during each transition from one generation to another in order to progressively decrease the fitness value until the predefined number of generations is reached. In this work, the fitness value was calculated using two different fitness functions and was taken as the classification loss of the SVM or kNN classifier. The population size was initially 5 chromosomes in which each gene was a feature that might be included (1) or rejected (0). The probability that it would mutate was 10% and that of a cross-over was 80%.

### 4.4. Classification

In this study, the dataset was divided into 2 different subsets. The dataset was randomly split into 80% training and 20% testing subsets using stratified sampling to maintain class proportions between BBB+ and BBB− compounds.

- For training, 80% of the dataset was used, and a set of feature vectors with known output was employed to build the classifier.
- The remaining 20% was used as the testing set: A classifier was tested by predicting the outputs of a test set and comparing the predicted results to the actual ones. This step is important to evaluate the performance of any classifier used. Once both sets were ready, the following types of classifiers were applied for performance comparison on the different classifiers: SVM [17] (linear SVM and using polynomial and radial basis function (RBF) kernels), LDA [18] and quadratic discriminant analysis (QDA) [19], and kNN. These classifiers were chosen based on their prevalent use in the literature regarding BBB permeability prediction and their diversity in algorithmic approach: SVM for handling non-linear separation, LDA and QDA for modeling linear and quadratic class boundaries, and kNN as a non-parametric baseline.

Additionally, an Artificial Neural Network (ANN) model [20,21] was implemented using a feed-forward architecture with one hidden layer composed of 30 neurons and trained via backpropagation. The ANN model achieved an accuracy of 94.6%, demonstrating its strong ability to capture complex, non-linear relationships between molecular descriptors and BBB permeability. This performance further improved to 96.23% when combined with feature selection techniques such as Sequential Feature Selection (SFS) and Genetic Algorithms (GA). The successful use of ANN in this context reflects its inspiration from biological neural systems and its capacity to learn predictive patterns in high-dimensional drug data, reinforcing its utility as a powerful tool in early-stage drug discovery pipelines.

### 4.5. Performance Evaluation

The performance of each classifier was individually evaluated by using the confusion matrix (insert reference here) technique, which allows us to compute the following parameters:

- The sensitivity (SE), which reflects the capacity of the classifier to detect BBB+ drugs in the entire dataset;
- The positive predictive value (PP), which expresses its ability not to deem non-crossing drugs as BBB+;
- The negative predictive value (NP), which reflects its ability not to deem crossing drugs as BBB−;
- The specificity (SP), which expresses the ability of the model to detect BBB- drugs in the dataset;
- The overall accuracy (ACC), which expresses the total true predictions over the total number of prediction.

The receiver operating characteristic (ROC) [22] curve was also applied in our study.

## 5. Conclusions

This work involves the application and comparison of two different types of feature selection algorithms on a CNS-based dataset. The results show that the GA method resulted in a more pronounced improvement in prediction accuracy than SFS. The best classifiers obtained after performing the GA yielded an accuracy of 96.23% and had a relatively good balance between predicting BBB+ drugs to BBB- ones. While this computational approach offers substantial efficiency in the drug discovery pipeline by reducing time and cost, it is not without limitations. The risk of false negatives could lead to missed opportunities for therapeutic development, potentially impacting patient outcomes. Therefore, these models should serve as decision-support tools and must be complemented by rigorous in vitro and in vivo validation to ensure patient safety and drug efficacy.

## Figures and Tables

**Table 1:** Overall accuracy computed prior to applying feature selection.

SVM (Linear)	SVM (RBF)	SVM (Polynomial)	LDA	QDA	kNN	ANN
**93.28**	93.35	93.03	92.72	92.78	93.10	94.6%

**Table 2:** Selected features using the SFS algorithm.

Classifier Used	Features Chosen
**SVM (linear)**	PSA, logP, HD, pKa (strongest acidic), NRB
**SVM (RBF)**	HD, HA, pKa (strongest acidic)
**SVM (polynomial)**	HD, HA, NRB
**LDA**	All but the HA
**QDA**	MW, PSA, HD, pKa (strongest acidic), pKa (strongest basic)
**k-NN**	All but pKa (strongest basic) and NRB
**ANN**	MW, PSA, HD, pKa (strongest acidic), NRB

**Table 3:** Summary of the overall accuracy obtained with each classifier after applying SFS and GA.

	SVM (Linear)	SVM (RBF)	SVM (Polynomial)	LDA	QDA	kNN	ANN
Without feature selection	93.28%	93.35%	93.03%	92.72%	92.78%	93.10%	94.6%
Backward SFS	94.67%	92.79%	88.4013%	93.73%	94.98%	94.36%	95.51%
GA: KNN based Fitness function	94.67%	94.04%	84.01%	94.36%	96.23%	92.79%	95.89%
GA:SVM based	93.73%	94.98%	96.23%	94.98%	95.62%	93.42%	96.04%

**Table 4:** Comparison of the performance of the best two classifiers.

	QDA + GA (kNN Based Fitness Function)	SVM + GA (SVM Based Fitness Function)
**True+**	256	255
**True−**	51	52
**False+**	11	4
**False−**	1	8
**SE**	95.88%	98.45%
**PP**	99.61%	96.95%
**SP**	98.07%	86.67%
**NP**	82.25%	92.85%
**ACC**	96.23%	96.23%

## Data Availability

The original contributions presented in this study are included in the article. Further inquiries can be directed to the corresponding author.

## Abbreviations

ANN: artificial neural network, BBB: blood–brain barrier, CNS: central nervous system, DT: decision tree, GA: genetic algorithm, HA: number of hydrogen bond acceptors, HD: number of hydrogen bond donors, kNN: k-nearest neighbor, LDA: linear discriminant analysis, logBB: logarithm of the ratio of the concentration in brain to that in blood, MLP: multilayer perceptron, MW: molecular weight, NRB: number of rotatable bonds, PR: permeation rate, RBF: radial basis function, RF: random forest, SFS: sequential feature selection, SVM: support vector machine.

## References

[1] Zlokovic B.V. (2008). The Blood-Brain Barrier in Health and Chronic Neurodegenerative Disorders. Neuron.

[2] Banerjee J., Shi Y., Azevedo H.S. (2016). In vitro blood–brain barrier models for drug research: State-of-the-art and new perspectives on reconstituting these models on artificial basement membrane platforms. Drug Discov. Today.

[3] Vastag M., Keseru G.M. (2009). Current in vitro and in silico models of blood-brain barrier penetration: A practical view. Curr. Opin. Drug Discov. Dev..

[4] Saraiva C., Praça C., Ferreira R., Santos T., Ferreira L., Bernardino L. (2016). Nanoparticle-mediated brain drug delivery: Overcoming blood–brain barrier to treat neurodegenerative diseases. J. Control. Release.

[5] Muehlbacher M., Spitzer G.M., Liedl K.R., Kornhuber J. (2011). Qualitative prediction of blood–brain barrier permeability on a large and refined dataset. J. Comput. Aided Mol. Des..

[6] Kunwittaya S., Nantasenamat C., Treeratanapiboon L., Srisarin A., Isarankura-Na-Ayudhya C., Prachayasittikul V. (2013). Influence of logBB cut-off on the prediction of blood-brain barrier permeability. Biomed. Appl. Technol. J..

[7] Castillo-Garit J.A., Casanola-Martin G.M., Le-Thi-Thu H., Barigye S.J. (2017). A simple method to predict blood-brain barrier permeability of drug-like compounds using classification trees. Med. Chem..

[8] Wang Z., Yang H., Wu Z., Wang T., Li W., Tang Y., Liu G. (2018). In Silico Prediction of Blood–Brain Barrier Permeability of Compounds by Machine Learning and Resampling Methods. ChemMedChem.

[9] Singh M., Divakaran R., Konda L.S.K., Kristam R. (2019). A classification model for blood brain barrier penetration. J. Mol. Graph. Model..

[10] Adenot M., Lahana R. (2004). Blood-Brain Barrier Permeation Models: Discriminating between Potential CNS and Non-CNS Drugs Including PGlycoprotein Substrates. J. Chem. Inf. Comput. Sci..

[11] Zhang D., Xiao J., Zhou N., Zheng M., Luo X., Jiang H., Chen K. (2015). A Genetic Algorithm Based Support Vector Machine Model for Blood-Brain Barrier Penetration Prediction. Biomed Res. Int..

[12] Yuan Y., Zheng F., Zhan C. (2018). Improved Prediction of Blood–Brain Barrier Permeability Through Machine Learning with Combined Use of Molecular Property-Based Descriptors and Fingerprints. AAPS J..

[13] Brito-Sánchez Y., Marrero-Ponce Y., Barigye S.J., Yaber-Goenaga I., Morell Perez C., Le-Thi-Thu H., Cherkasov A. (2015). Towards Better BBB Passage Prediction Using an Extensive and Curated Data Set. Mol. Inform..

[14] Miao R., Xia L.Y., Chen H.H., Huang H.H., Liang Y. (2019). Improved Classification of Blood-Brain-Barrier Drugs Using Deep Learning. Sci. Rep..

[15] Zhao Y.H., Abraham M.H., Ibrahim A., Fish P.V., Cole S., Lewis M.L., de Groot M.J., Reynolds D.P. (2007). Predicting Penetration Across the Blood-Brain Barrier from Simple Descriptors and Fragmentation Schemes. J. Chem. Inf. Model..

[16] Robu R., Holban S. A genetic algorithm for classification. Proceedings of the 2011 International Conference on Computers and Computing (ICCC’11).

[17] Ben-Hur A., Ong C.S., Sonnenburg S., Schölkopf B., Rätsch G. (2008). Support vector machines and kernels for computational biology. PLoS Comput. Biol..

[18] Linear Discriminant Analysis. http://www.saedsayad.com/lda.htm.

[19] Linear & Quadratic Discriminant Analysis·UC Business Analytics R Programming Guide. https://uc-r.github.io/discriminant_analysis.

[20] Artificial Neural Network. https://www.saedsayad.com/artificial_neural_network.htm.

[21] Yippy A Beginner’s Guide to Neural Networks and Deep Learning. https://skymind.ai/wiki/neural-network.

[22] Park S.H., Goo J.M., Jo C. (2004). Receiver Operating Characteristic. (ROC) Curve: Practical Review for Radiologists. Korean J. Radiol..

